# Study protocol: a double blind randomised control trial of high volume image guided injections in Achilles and patellar tendinopathy in a young active population

**DOI:** 10.1186/s12891-017-1564-7

**Published:** 2017-05-22

**Authors:** Robert M. Barker-Davies, Alastair Nicol, I. McCurdie, James Watson, Polly Baker, Patrick Wheeler, Daniel Fong, Mark Lewis, Alexander N. Bennett

**Affiliations:** 1 Academic Department of Military Rehabilitation, DMRC Headley Court, Epsom, Surrey KT18 6JW UK; 2 School of Sport, Exercise and Health Sciences, National Centre for Sport and Exercise Medicine-East Midlands, Loughboruough University, Leicestershire, LE11 3TU UK

## Abstract

**Background:**

Chronic tendinopathy is a significant problem particularly in active populations limiting sporting and occupational performance. The prevalence of patellar tendinopathy in some sports is near 50% and the incidence of lower limb tendinopathy is 1.4% p.a. in the UK Military. Management includes isometric, eccentric, heavy slow resistance exercises and extracorporeal shockwave therapy (ESWT). Often these treatments are inadequate yet there is no good evidence for injection therapies and success rates from surgery can be as low as 50%.

High Volume Image Guided Injection (HVIGI) proposes to strip away the neovascularity and disrupt the nerve ingrowth seen in chronic cases and has shown promising results in case series. This study aims to investigate the efficacy of HVIGI in a randomised controlled trial (RCT).

**Methods:**

RCT comparing 40ml HVIGI, with or without corticosteroid, with a 3ml local anaesthetic sham-control injection. Ninety-six participants will be recruited. Inclusion criteria: male, 18–55 years old, chronic Achilles or patellar tendinopathy of at least 6 months, failed conservative management including ESWT, and Ultrasound (US) evidence of neovascularisation, tendon thickening and echogenic changes. Outcome measures will be recorded at baseline, 6 weeks, 3, 6 and 12 months. Primary outcome measures include The Victoria Institute of Sport Assessments for Achilles and patellar tendinopathy (VISA-A and VISA-P) and VAS pain. Secondary outcome measures include Modified Ohberg score, maximum tendon diameter and assessment of hypoechoic appearance on US, and Functional Activity Assessment.

**Discussion:**

Despite previous interventional trials and reviews there is still insufficient evidence to guide injectable therapy for chronic tendinopathy that has failed conservative treatment. The scant evidence available suggests HVIGI has the greatest potential however there is no level one RCT evidence to support this. Investigating the efficacy of HVIGI against control in a RCT and separating the effect of HVIGI and corticosteroid will add high level evidence to the management of chronic tendinopathy resistant to conservative treatment.

**Trial Registration:**

EudraCT: 2015-003587-36 3 Dec 2015

## Background

Lower limb tendinopathy is a significant problem especially in the sporting population. The reported incidence increases from 1.85/1000 p.a. in the Achilles amongst the general population to 7.4% per month in marathon runners [1, 2]. In the patella prevalence is 10–15% in volleyball and rugby players and up to almost 50% in masters athletes [3–5]. Military personnel by the nature of their work are a physically active and sporting population. From the UK military Primary Care database incidence of lower limb tendinopathy is estimated at 1.4% p.a. representing a significant cause of morbidity. This represents a significant burden to the Armed Forces in terms of occupational and operational effectiveness. The unpredictable nature of tendinopathy by virtue of its poor correlation between pain, function and pathological stage adds uncertainty to workforce planning and career development for the individuals affected.

The tendinitis model in use up to the 1990s has been superseded [6]. A continuum model for tendinopathy whereby abnormal load is seen as the primary cause and optimal load management is seen as the primary treatment is now well established and accepted [7]. This model described by Cook and Purdam [8] based on histological findings provides the framework on which current best practice is designed and areas for further research are targeted. There is increased proteoglycan, ground substance and collagen separation with cells taking on a more chondrotic shape but absence of typical inflammatory mediators on traditional histology. Neovascularisation occurs as the pathology progresses and is thought to be associated with nerve ingrowth and pain. With this change of understanding many treatments have been tried, some with more success and better evidence than others.

Eccentric Loading Exercise *(EL)* is a well-established treatment in Achilles tendinopathy and has been developed successfully to treat other tendinopathies [9, 10]. Early trials by Alfredson [11, 12] were in older patients (mean age = 44) with a chronicity that would place them in the degenerate phase of Cook and Purdam’s continuum. Unmodified EL is not effective for insertional tendinopathy [13]. Heavy Slow Resistance Exercise (HSR*)* is an alternative to EL. Kongsgaard et al’s [14] RCT investigating patellar tendinopathy showed similar results for HSR and EL in terms of pain and function. HSR also yielded increased collagen network turnover and greater satisfaction than EL in a younger population (mean age = 32).

Extracorporeal Shockwave Therapy (ESWT) showed initially promising results in supraspinatus, elbow and heel tendinopathies [15]. Furia’s [16] trial of 68 patients showed superior results with ESWT compared to traditional therapy controls in insertional Achilles tendinopathy. Wang [17] et al. reported impressive results in favour of ESWT v. control in patellar tendinopathy with statistically significant differences in VISA-P scores at 2–3 year follow up. ESWT has been concluded in reviews by Wiegerinck [18] and van Leeuwen [19] to be a safe effective treatments for insertional Achilles and patellar tendinopathies respectively. It should be noted Zwerver [20] found no effect of ESWT in an RCT of 62 participants with patellar tendinopathy. Though they conclude the continued sports participation of mostly reactive to disrepair stage tendinopathies treated could explain the lack of effect and that ESWT may still be effective.

Unfortunately a number of patients still fail to respond to EL, HSR or ESWT. What to do next is a clinical dilemma with insufficient evidence to guide. Surgery remains an option but with success rates as low as 50% [21] there is a desire to find safe effective treatment that bridges the gap from conservative management.

High Volume Image Guided Injection (HVIGI) has been proposed to remove neovascularisation and disrupt nerve ingrowth [22]. Whilst there is no universally accepted pain model in tendinopathy there are well established mechanisms for associating angiogenesis with neoneuralisation including shared genetic pathways and neurotrophic effects of Vascular Endothelial Growth Factor (VEGF) [23]. Increased substance P positive nerve fibres may provide a nociceptive stimulus coupled with decreased pain modulation by noradrenaline [24]. Prolonged expression of substance P may stimulate the hypercellularity and altered morphology of tenocytes as well as stimulating endothelial cells [25]. It is anticipated a shearing or compressive effect of HVIGI by using the neovessels as a target area may achieve disruption of these nerve fibres. Such mechanical damage may act synergistically with other mechanisms for example neurotoxicity and vasoconstriction caused by the initial local anaesthetic bolus [26].

Table 1 outlines the HVIGI studies to date. The studies indicate positive results but all lack control groups and causality of benefit cannot be identified from these study designs. There is variation in duration of follow up and the use of corticosteroid (CS) making results of this group of studies difficult to interpret.

**Table 1:** Summary of HVIGI studies

Study	Intervention	Control	+/− VAS Pain	+/− Function eg VISA-A/P
Achilles tendinopathy				
Chan 2008 [68]	*N* = 30 10ml 0.5% Bupivacaine 25mg Hydrocortisone 40ml N Saline	No control	*N* = 21 @4w -51mm	*N* = 21 @4w VISA-A +31.4
Humphrey 2010 [69]	*N* = 11 10ml 0.5% Bupivacaine 25mg Hydrocortisone 40ml N Saline	No Control		@3w VISA-A +38 (*P* < 0.001) NeoVasc reduced 3 to 1.1 Tendon diam 8.7 to 7.6mm
Restighini 2012 [70]	*N* = 32 5ml 1% Lidocaine 25mg Hydrocortisone Up to 40ml N Saline	No Control	@4w -34mm @3m -37mm	@4w VISA-A +26.5 @3m VISA-A +28.7 NeoVasc reduced 2 to 0.2
Maffuli 2013 [71]	*N* = 94 10ml 0.5% Bupivacaine 25mg Aprotinin up to 40mls N saline	No Control		@12m VISA-A +32.9 (*P* = 0.003)
Wheeler 2014 [72]	*N* = 16 10ml 1% Lidocaine 40ml Saline	No Control	@347d −6.1/10 (*P* = 0.001)	@347d VISA-A + 41 (*P* = 0.001)
Wheeler 2016 [73]	*N* = 34 10ml 1% Lidocaine + 40ml Saline V 10ml 1% Lidocaine + 20ml Saline	No Control	@281d −4.6/10 (*P* < 0.01) 50ml Group	@281v271d VISA-A +33.4 and + 6.94 in 50ml and 30ml group respectively (*P* = 0.002)
Patellar tendinopathy				
Crisp 2008 [74]	*N* = 9 10ml 0.5% Bupivacaine 25mg Hydrocortisone 40ml N Saline	No control	@2w Sig. improve.	@9m VISA-P +22
Morton 2014 [22]	*N* = 20 10 ml 0.5% Bupivacaine 25mg hydrocortisone 30ml N saline	No Control		@12w–9m VISA-P +19 (*P* < 0.01)
Maffuli 2016 [75]	*N* = 44 10ml 0.5% Bupivacaine 62500 IU Aprotinin 40ml N saline	No Control	@15m -63mm (*P* = 0.01)	@15m VISA-P +29.3 (*P* = 0.003)

*VISA-A* Victoria Institute of Sport Assessment-Achilles, *VISA-P* Victoria Institute of Sport Assessment-Patella, *VAS* Visual Analogue Scale, *NeoVasc* Grade of Neovasculatity, *IU* International Units, *m* months, *w* weeks, *d* days

The primary objective of this study therefore is to identify through a RCT the effectiveness of HVIGI using injectable saline (40ml) in combination with local anaesthetic with/without injected CS compared to a sham-control injection in patients with chronic tendinopathy unresponsive to conservative therapies.

The hypotheses are that HVIGI in both the presence and absence of CS will be superior to control and that HVIGI without CS will be non-inferior to HVIGI with CS.

## Methods/Design

### Double blind Randomised Control Trial (RCT)

- Low volume (3ml) US guided subcutaneous Local Anaesthetic (LA) sham-control injection.
  or
- HVIGI: Saline + Local Anaesthetic (LA). Total volume 40ml.
  or
- HVIGI: Saline + LA + CS. Total volume 40ml.

#### Participants inclusion/exclusion criteria

##### Inclusion criteria

Male patients with either Achilles or patellar tendinopathy of at least 6 months chronicity who have failed best practice management (Table 2) including EL, HSR and ESWT aged 18–55 and US evidence of neovascularisation, tendon thickening and echogenic changes will be included in the study. Patients must be medically unfit for their full military role. The level of function relating to military medical grading relates to the patient’s individual role. The relationship between this and VISA scores will be explored as part of this research. The diagnostic guide used for initial diagnosis in primary care is provided in Table 3. Diagnosis is confirmed by a Sport and Exercise Medicine Consultant with the additional US criteria above.

**Table 2:** Defence rehabilitation Achilles and Patellar tendinopathy best practice management pathway

Tendon loading stage	Type of contraction	Reps/Sets/Holds	Aim of phase	Progression criteria	
				Load tolerance tests	Outcome measures
Stage 1 – Low Load Daily exercise	Isometric (may avoid painful range initially)	5x 15–60 s holds Repeated 4x daily 5 days/week	Inhibit cortical interpretation of pain. Tendon adaptation to load	Able to move body weight double leg with pain less than 3/10 eg, squat, calf raise	Numeric Rating Scale or Visual Analogue Scale On first waking On activity After activity VISA A/P 6 weekly intervals
Stage 2 – Medium Load Alternate days with Stage 1	Eccentric/Concentric (may avoid painful range initially)	3x 10–12 3 s Eccentric / 3 s Concentric	Inhibit cortical interpretation of pain Musculo-tendinous adaptation to load.	Able to move body weight single leg with pain less than 3/10, eg single leg squat/single leg calf raise	
Stage 3 – High Load Every third day with Stage 1 and 2 on days inbetween	Eccentric/ Concentric Heavy Slow Resistance	3x15 (reduced progressively as weight increased) 3 s Eccentric /3 s Concentric 60% 1RM (3x15 reps) progressed to 85% 1RM (3x6 reps) in week 12	Musculo-tendinous adaptation to load through controlled movements	Able to perform low level plyometric test with less than 3/10 pain, eg repeated small hop or split jump	
	Eccentric Loading	3x15 (double leg, single leg, weighted) Up to 10 s eccentric phase			
Stage 4 – High Load In place of Stage 3 loading, with Stage 1 and 2 inbetween	Plyometric	Restricted to 60 foot contacts when introduced. eg. 3x10 Forward hops 3x10 Single leg hops Increased to 80 foot contacts	Elastic loading of Musculotendinous unit.	Able to perform high level plyometric test e.g. repeated maximal hop and multidirectional hop.	

*RM* Repetition Maximum, *VISA-A* Victoria Institute of Sport Assessment-Achilles, *VISA-P* Victoria Institute of Sport Assessment-Patella

**Table 3:** Diagnostic guide

	Achilles tendon (inc Insertional)	Patella tendon
Causative factors	Common in running sports and may have been precipitated by a change in training load (volumes, duration or intensity).	Common in running, tabbing and jumping sports and may have been precipitated by a change in training load (volumes, duration or intensity).
Pain site	Localised tenderness in mid portion of Achilles (2–6cm from insertion) or at insertion.	Pain at inferior pole of patella.
Pain pattern	Pain may occur at start of activity and then ease, or may increase with duration of activity. Pain after period of rest or on waking.	Pain may occur at start of activity and then ease, or may increase with duration of activity. Pain after period of rest or on waking.
Palpation findings	Localised tenderness in mid portion of Achilles (2–6cm from insertion) or at insertion.	Focal pain on palpation.
Provocation and Load Tolerance Tests	Calf raise Small hop Repeated hop Repeated hop Multidirectional Maximal forward hop	Leg extension Single leg decline squat Small hop Split jump Maximal hop

##### Exclusion criteria

Females, patients with a concurrent alternate lower limb diagnosis, those on anticoagulant medication or having had previous tendon surgery or injection to the affected limb will be excluded. Plantaris involvement in Achilles tendinopathy will be treated as an alternate lower limb diagnosis as this is associated with resistance to conservative treatment and surgery may be more appropriate [27]. Tears and partial tears will also be treated as alternate diagnoses.

### Follow up

Outcome measures will be taken at baseline and at 6 weeks and 3, 6 and 12 months follow up.

### Primary endpoint/outcome

- The Visual Analogue Scale (VAS) for pain on day of review at 6 months.
- Validated Victoria Institute of Sport Assessment-Achilles (VISA-A) [28] and Victoria Institute of Sport Assessment-Patella (VISA-P) [29] scores for pain and function at 6 months.

### Secondary endpoints/outcomes

- VAS for pain on day of review at 6 weeks, 3 and 12 months.
- VISA-A and VISA-P scores for pain and function at 6 weeks, 3 and 12 months.
- The degree of neovascularisation measured using the modified Ohberg Score [30] at 6 weeks, 3 and 6 months. Scoring will be made by one of the 3 operators delivering injections usually PB and occasionally AN, and IM if required. Operators have undertaken training in Ohberg scoring and demonstrated inter and intra-rater reliability with Intraclass Correlation Coefficients of 0.89 and 0.92 respectively. This was achieved using dynamic US for inter-rater and archived images for intra-rater reliability in a participant subset separate from this RCT of 16 symptomatic tendons of which 12 had neovascularity.
- The Functional Activity Assessment (FAA) score which is validated in the military population [31] at 6 weeks, 3, 6 and 12 months.
- Strength and balance testing as assessed by study physiotherapist (JW) at 6 weeks, 3 and 6 months. This includes the small knee bend score [32] and 5 repetition maximum calf raise and leg extension for Achilles and patellar tendinopathies respectively (Table 4). Adherence to the rehabilitation programme will also be scored using a percentage measure. This is self-reported by the participant using a logbook. The study physiotherapist multiplies out the number of sets and repetitions recorded then divides by the total number prescribed using this proportion multiplied by 100 to arrive at the final score.

**Table 4:** 5 Repetition maximum testing (5RM)

Step	Protocol
1	Patient to adopt relevant exercise position on specific machine – a or b below: a. Heel raise on leg press through full dorsi/plantarflexion range (Achilles) b. Knee extension 0–90° (Patella)
2	Estimate a low warm up load that will allow the participant to complete 10 repetitions ensuring they use the full range available
3	Provide a rest period (1–2 min)
4	Estimate a load that will allow the participant to complete 5 repetitions
5	Participant attempts 5 repetitions
6	Provide a rest period (1–2 min)
7	If successful increase load and repeat test
	Repeat steps 5–7 until participant unable to complete 5 repetitions or pain reaches 4/10 or more.
8	Record final load that the participant completed for 5 repetitions

The 5RM provides an objective marker for load capacity of a certain muscle group. Limitation of this test is that weight can only be added by increments that the machine allows (5Kg/heel raise, 2.5Kg/knee extension). Machine is standardised for every test. Decision to use machines rather than test with free weights is primarily based on safety. Ideally Isokinetic testing would be preferred but is not available

#### Procedure (Table 5)

**Table 5:** Patient timeline (Spirit Figure)

	Study period						
	Enrolment	Allocation	Post-allocation				Close-out
Timepoint	Referral from RRU	First clinic	6w	3m	6m	12m (remote)	
Enrolment:		X					6m clinic, 12m remote
Eligibility screen	X	X					
Informed consent	Written info sent	X					
Allocation/Random		X					
Interventions:							
*RCT Injection*		X					
*Progressive Exercise Loading Programme*		X	X	X	X	X	
Assessments:							
*Epidemiology Questionnaire*		X					
*VISA, VAS, FAA,*		X	X	X	X	X	
*USS, Ohberg*		X	X	X	X		
*Strength/ Small Knee Bend*		X	X	X	X		
*Adherence to Rehabilitation Programme*		X	X	X	X		

As testing and interventions outlined in this study may be delivered to eligible participants at the first appointment information will be sent in advance. This will be done by post providing information sheets. Eligible patients will be asked to complete the informed consent form at the first appointment with the consultant performing the intervention. Ineligible patients will continue to be treated as per current guidelines as will those that decline study participation.

During the follow up period all participants will be advised to continue with a home exercise programme. Physiotherapy treatment will continue at primary care and Regional Rehabilitation Unit (RRU) level as required. Oversight of this will be maintained by the lead physiotherapist at Defence Medical Rehabilitation Centre (DMRC) Headley Court by providing instruction to primary care and RRU physiotherapists directly to ensure standardisation of post intervention physiotherapy. All participants will be progressed along an incremental pathway of rest, isometric, isotonic, EL/HSR and then sport specific activity including plyometrics as per Table 2. Participants will be asked to refrain from seeking alternative treatments during the trial and to complete an exercise logbook specific to this study.

#### Randomisation and blinding procedures

One hundred and eight participants (see power calculation below) will be allocated in equal proportions across the 3 intervention arms. 12 batches of 9 envelopes numbering 108 envelopes in total with 36 pertaining to each arm of the study will be prepared and secured in a locked container held centrally at DMRC. In this manner should the recruited numbers differ participants will be randomly allocated whilst the proportion of numbers across all 3 arms will be equal throughout the data collection phase.

Once participant eligibility and informed consent are confirmed, an independent administrator will open one of the batched envelopes to assign the participant to one arm of the trial. The treating clinicians including physiotherapists and consultants delivering injection interventions will be blinded to which of the three injections they deliver. The Chief Investigator will be informed of which participants are involved in each trial arm and responsible for preparation of injection syringes checked by a nurse or other doctor not involved in the trial. The reception staff will direct participants to a separate area in the waiting room where they will be able to complete VAS, VISA and FAA questionnaires in private. The reception staff will check the questionnaires for basic errors (eg a missed page) before filing this in a locked store. The study administrator will collate these outcome measures on a spreadsheet designed by the Chief Investigator this will be kept in a locked store and backed up electronically in a password protected Microsoft Excel file on the Defence computer network. The Chief Investigator will store hard copies of the outcome measures in the locked Academic Department of Military Rehabilitation (ADMR) store and update a second outcomes spreadsheet so that double data entry is achieved. A separate store with a list containing the consecutive trial numbers 1–108 ascribing those numbers to patient notes numbers and which trial arm they are in will be maintained by the Chief Investigator and secured in a locked store within the ADMR. A second copy of this list will be continually updated and provided to the Independent Medical Officer who will store this list in a locked store. This is so that in case of emergency either the Chief Investigator or Independent Medical Officer could be contacted to confirm which treatment a participant had been given. The DMRC Pharmacy will also retain hard copies of individual prescriptions as well as the prescriptions log.

Fully blinding the control injection arm to the clinician is not possible due to the small volume of injectate (3ml of LA) and difference in procedure; neovessels will not be targeted under US guidance. The two treatment arms will be fully blinded to both clinician and participants, as the volumes delivered will be equal and technique identical. The steroid to be used does not appear opaque in the syringe maintaining clinician blinding. Clinicians and participants will be shielded from syringe preparation. To assess blinding at trial completion participants will be asked if they think they were randomised to any individual trial arm, if they think they received either a HVIGI or control or if they don’t know which injection they received.

#### Interventions

Participants will be randomised to receive one of the 3 injections below. Prior to the intervention the clinician (PB) performs a diagnostic US. As well as other factors described in the secondary outcomes neovascularity is identified. Under US guidance a 21G needle is placed between the anterior aspect of the Achilles tendon and Kager’s fat pad or posterior aspect of the patella tendon and Hoffa’s fat pad at the area of maximum neovascularisation. Connecting tubing attached with a luer lock to a 10ml syringe is then connected to the needle. Under instruction of the clinician the assistant then delivers the contents of the syringe to separate the fat pad from the tendon. During the procedure the needle is re-orientated to ensure maximum separation of the fat pad from the tendon. A further 10ml syringe then replaces the empty syringe until 40ml of injectate has been delivered. In each case the first syringe will contain the LA with/without an additional 0.25ml of CS. Three 10ml syringes of saline will then follow, followed by a fifth syringe of 2.75–3ml (depending on whether or not the first syringe contained steroid) to allow for the blind space in the connecting tubing. In all injections an aseptic technique will be used. After the procedure the clinician will rescan the tendon ensuring neovascularity has disappeared.

The Chief Investigator will prescribe in accordance with current policy approved by the DMRC Medicines Management Committee by hard copy prescription. A statement will be entered in the patient notes with appropriate contact details. All participants will receive a card detailing that they may have been treated with any of the combinations of medications below:

- Low volume (3ml) Peritendinous Local Anaesthetic (LA) Control:
  Bupivacaine 0.5%, 3ml = 15mg. **Total Volume 3ml**
  Brand: Marcain Polyamp (Astra Zenica) 0.5%
- HVIGI Saline (to obliterate neovascularisation on US) + Local Anaesthetic (LA).
  Bupivacaine 0.5%, 10ml = 50mg + Saline 30ml. **Total Volume 40ml**
  Brand: Marcain Polyamp (Astra Zenica) 0.5% + Sodium Chloride (Hameln Pharmaceuticals) 0.9%
- HVIGI Saline (to obliterate neovascularisation on US) + LA + Corticosteroid (CS).
  Bupivacaine 0.5%, 10ml = 50mg + Hydrocortisone 100mg/ml 0.25ml = 25mg + Saline 29.75ml **Total Volume 40ml**
  Brand: Marcain Polyamp (Astra Zenica) 0.5% + Efcortesol (Amdipharm UK Limited) 100mg/ml + Sodium Chloride (Hameln Pharmaceuticals) 0.9%

After completion of the trial details of which combination above was prescribed will be added to the patient notes.

## Study setting

The study will take place entirely at DMRC Headley Court. This is the MOD’s tertiary referral centre for Sport Exercise and Rehabilitation Medicine. Patients are referred to Headley Court as part of routine care.

### Recruitment

RRU doctors have been briefed and provided with additional information to have an awareness of this trial and may suggest referral to DMRC Headley Court once the physical therapies in the current guidelines and ESWT have been tried without success. They may mention that a trial is taking place.

### Patient identification

The trial will be mentioned by RRU staff to potential participants and further information will be provided. Once a referral has been made the patient will be sent written information and a copy of the consent form so that they have a minimum of 24 h to consider the information before attending DMRC. It will be stressed that if patients do not wish to participate in the trial they will not be prejudiced and will be treated in line with current best practice. They will still be welcomed to attend DMRC appointments.

### Screening

Patients will be screened as per their normal routine care for a tertiary referral to DMRC Headley Court. They will have a 45-min initial appointment with one of the research team consultants. This will include reviewing their medical history and checking parameters such as weight, BMI, pulse and blood pressure. No more invasive tests are required.

### Blinding and data access

The Chief Investigator the Independent Medical Officer and DMRC Pharmacy will have access to the list of which participant corresponds to which trial number and which group they have been randomised to.

The treating clinicians will have access to the clinical record and images on the US machine.

The study administrator, Chief Investigator and ADMR Manager (Data Monitor) will have access to the original copies of outcome measure data (Case Report Forms) and collated results spreadsheets. Should for reasons of lack of participants the trial need to be declared complete short of the number identified by power calculation this decision will be taken in conjunction with MOD and Loughborough University supervisors (AB, DF, ML and PW).

### Withdrawal criteria

In accordance with the Declaration of Helsinki participants will be free to withdraw at any time. This trial requires that a single injection only is delivered on the day of consenting to the trial. Compliance and drop out in this respect is therefore not anticipated to be a problem.

It is possible that participants may perceive having to travel and attend follow-up appointments an inconvenience. Under those circumstances further options for gaining follow up data include its collection at RRU and failing that primary outcome measures could be assessed remotely.

Should the participant no longer wish to participate in the physical elements of the rehabilitation, which is current standard practice they are of course free to do so. A 10% drop out rate has been allowed for (see below).

### Sample size

The effect size *f* has been calculated along with the overall predicted sample size calculation using GPower (University of Dusseldorf, GER) [33]. The Minimum Clinically Important Difference (MCID) in the VISA-P score in athletes with patellar tendinopathy who underwent conservative management was calculated by Hernandez-Sanchez [34] to be 12.6 (*n* = 90 baseline VISA-P = 50.1 (SD = 18.4). The effect size *f* of 0.3228096 was combined with an α-level of 0.05 and power of 0.8 in a priori power calculation for an ANOVA, fixed effects, omnibus one way test predicting an initial sample size of 96. The calculation was repeated so that addition of a suspected covariate (percentage adherence to the standardised rehabilitation protocol) could be considered in ANCOVA again a sample size of 96 was predicted. Further calculations were made to allow using repeated measures, between factors ANOVA assuming correlation of 0.5 among repeated measures this returned a smaller total sample sizes of 75 for primary outcome measures (baseline and 6 month follow-up) and 60 for secondary outcome measures (baseline, 6 weeks, 3,6 and 12 month follow-ups). 108 participants with either Achilles or patellar tendinopathy will be recruited to allow for a 10% drop out rate.

### Statistical analyses

Demographic data over the 3 RCT groups will be compared. This will include the risk factors to be analysed as part of a correlational study including age, obesity, smoking, blood group, hypertension and family history. Parametric assumption checks will be made and one-way ANOVA used to check for statistical differences that may have occurred by chance at baseline. Where necessary ANCOVA will be used to correct differences across the groups. The adherence to rehabilitation score will be considered as a covariate.

The primary outcome data will be compared between groups at baseline and 6 months. Individual outcome measures for each group will be analysed to check parametric assumptions. For primary and secondary outcome measures a repeated measures, between factors ANOVA (time x intervention) will be used to look for significant difference. Groups will then be compared against each other using *post hoc* pairwise comparisons using SPSS software and effect sizes calculated.

A protocol deviation log is to be kept so that unanticipated trends can be analysed. The proportion of missing data will be reported with reasons where possible. Missing data will be handled using multiple imputation calculated in SPSS. A minimum of 5 imputations will be used. Results for completed cases will be compared to those based on multiple imputation and reported in accordance with the guidance proposed by Sterne et al. [35].

### Roles and responsibilities

The role of the Sponsor (Director of Defence Rehabilitation) is to: “Promote effective professional guidelines and support to medical rehabilitation providers. Develop, promote and co-ordinate the standardisation and delivery of best evidence clinical practice across Defence. To support medical rehabilitation in the operational environment in order to assist in the maintenance of fighting power. To provide an inspectorate role across Defence Medical Rehabilitation. To provide leadership for rehabilitation cadres, including: Rheumatology & Rehabilitation consultant, Sport & Exercise Medicine, Physiotherapy, Exercise Rehabilitation Instruction and other Allied Health Professional groups. Co-ordination, collection and analysis of medical intelligence and data related to rehabilitation.”

As the Director of Defence Rehabilitation (DDR) The Sponsor welcomes this research to further develop the DDR tendinopathy guidelines. The Sponsor can be contacted via email SGDPHC-DefRehab-DDR@mod.uk.

Given the nature of this research an independent trial steering committee has not been appointed. An Independent Medical Officer (IMO) has been appointed to act as a point of contact outside of the research team. The IMO is available for patients/participants and staff members who may have any questions or concerns that might not necessitate the significant event reporting process.

The Trial Management Group meets at least quarterly and consists of the authors listed above.

This protocol has been reviewed and approved by the: The Defence Medical Services Research Steering Group, The Surgeon General’s Higher Degree Board, The Dean of the School of Sport, Exercise and Health Sciences, Loughborough University, The Defence Science and Technology Laboratory’s Scientific Assessment Committee, MODREC with acceptance of this approval by LU ethics committee and the MHRA. Amendments suggested by these bodies have been incorporated into this protocol.

### Monitoring

An Independent Data Monitoring Committee (IDMC) has been appointed in accordance with National Research Ethics Service guidance [36] and the DAMOCLES study group recommendations [37]. Members are the clinical research manager of ADMR, the chair of the DMRC Medicines Management Committee, the DMRC Clinical Quality Manager and an Independent statistician. The Chief Investigator will analyse and make available accruing data, the protocol deviation log and significant event reports prior to the IDMC meeting 6 monthly. The ADMR manager will inspect all locked stores containing Case Report Forms and audit transfer to results spreadsheets monthly. As set out in its charter the IDMC makes recommendations to the Sponsor who retains executive decision-making on trial stopping.

### Safety reporting

All Significant Adverse Events and Suspected Unexpected Serious Adverse Reactions (SAEs/SUSARs) occurring from the time of written informed consent and start of trial treatment until 6 months post cessation of trial treatment and discharge from the care of DMRC Headley Court must be recorded on a reporting form and the Sponsor notified within 24 h of the research staff becoming aware of the event. Full details, duration, action taken, outcome, seriousness criteria, causality and expectedness will be collected. Any change of condition or other follow-up information should be made available to the Sponsor via the DMRC Clinical Quality Manager as soon as it is available or at least within 24 h of the information becoming available. Events will be followed up until the event has resolved or a final outcome has been reached.

All SAEs assigned by the CI or delegate (or following central review) as both suspected to be related to IMP-treatment and unexpected will be classified as SUSARs and will be subject to expedited reporting to the Medicines and Healthcare Products Regulatory Agency (MHRA). The Sponsor will inform the MHRA, the REC and the Sponsor of SUSARs within the required expedited reporting timescales. If any urgent safety measures are taken the CI/Sponsor they shall immediately and in any event no later than 3 days from the date the measures are taken, give written notice to the MHRA and the relevant REC of the measures taken and the circumstances giving rise to those measures.

### Governance and dissemination of results

In accordance with Good Clinical Practice (GCP) guidance personally identifiable information will only be available on the enrolment log which is kept in a locked store as part of the trial master file. All other source documents will be identified using the unique participant trial number and initials.

As described above only the study administrator, Chief Investigator and ADMR manager will have immediate access to the full dataset during the trial. The IDMC will be able to review accrued data 6 monthly and it is stipulated in their charter not to discuss these results prior to completion of the trial. In accordance with the Defence Rehabilitation – Transfer of Research Findings into Best Practice algorithm the Chief Investigator will complete the trial report. The report will then be forwarded to the Defence Director of Rehabilitation’s (DDR) Clinical Policy Committee (CPC) for consideration. The CPC Chair (DDR) will then instruct the Best Practice Guideline Chair to review the findings. The Best Practice Guideline Chair and Chief Investigator will together prepare updated guidelines for submission to the CPC for approval. The CPC will also recommend the original report be approved for publication. This manuscript will be subject to approval by The Surgeon General’s Director of Research and the Commanding Officer of DMRC Headley Court. Publication will be sought in a relevant peer review journal with as high an impact factor as possible. Loughborough University will be acknowledged for their support in this research. The Chief Investigator’s final report will also be submitted to Loughborough University in the format of a PhD thesis. The MOD retains ownership and rights to publish the data. Results in lay terms will be made available to participants. All reports are to be written up no later than 12 months following completion of the trial. The research team listed as authors to this protocol will be included as authors on research publications arising from this trial. The Chief Investigator will be first author. Professional medical writers will not be hired. Additional advice may be sought on the areas of statistics and biomechanical analysis and should significant contribution be made those parties listed as authors. The full protocol and participant level data set may be requested from the Chief Investigator. Trial participants in accordance with The Declaration of Helsinki 2013 will have access to any treatments shown to be beneficial at the end of the trial that remain appropriate for them on medical grounds. As the tertiary referral centre for Rehabilitation in the Armed Forces DMRC Headley Court will continue to care for these patients where appropriate. A research participant wishing to seek no-fault compensation should contact the DBR Common Law Claims & Policy (CLCP), Ministry of Defence, Level 1, Spine 3, Zone J, Whitehall, London, SW1A 2HB.

## Discussion

Kearney’s Cochrane review of all injection therapies in Achilles tendinopathy found insufficient evidence to recommend any individual therapy and recommended that injections should take place in a research setting [38]. Other than HVIGI covered above options include CS, Autologous Blood (AB), Platelet Rich Plasma (PRP), Dextrose and polidocanol.

Traditionally CS injection was commonplace but with the demise of the tendinitis model and case reports of tendon rupture [39, 40] its use has become less popular. Coombes et al’s [41] systematic review of RCTs found of the trials that reported adverse effects only one in 991 participants suffered tendon rupture following CS injection. They found CS to be effective in the short term but to have no benefit at intermediate or long term follow up. Kongsgaard et al.[14] examined the tissue biopsy samples post steroid injection and found mechanical properties to be unaffected.

Rees et al. [42] make a case for re-visiting inflammation. They highlight that more recent research techniques have demonstrated the presence of inflammatory mediators such as macrophage derived IL-1, COX-1, COX-2, IL-6, TGF-β and Substance-P. Whilst acknowledging that mechanical overload remains the primary driver initiating tendinopathy they call for emphasis on treatments that address these elements of inflammation. Therefore there may then still be a place for CS injection in combination with the evidence-based physical therapies discussed above. US guidance to confirm peritendinous rather than intratendinous injection may also mitigate risks further [43]. CS appears to be useful for short-term symptom improvement rather than the sustained improvement to the underlying pathology. A RCT investigating HVIGI treatment with or without CS has yet to be published.

Dextrose is proposed to cause a local inflammatory reaction and injected intratendinously. Dextrose has shown promising results in case series in patellar [44] and Achilles [45, 46] tendinopathy but is yet to be reported against control in these tendons. Although these studies report no adverse effects Knobloch highlights the potential for increased tendon hypoxia and infection due to the intratendinous placement of the agent [47]. In contrast polidocanol is injected peripheral to the tendon to sclerose the neovascularity thus mitigating these risks [48]. In patellar tendinopathy polidocanol has shown significant improvement against control in a RCT [49]. However in contrast to HVIGI both dextrose and polidocanol usually require repeat injections. In the case of polidocanol in Hoksrud’s RCT the mean number of injections, which are considered difficult to perform, was 3.6 ± 1.5 in the treatment group over 8 months. The control group (*n* = 16) was also crossed over to the intervention after 4 months at which stage the improvement in the treatment group was less than the MCID mentioned above [49].

AB/PRP injections in theory deliver an intratendinous stimulus of growth factors to correct failed healing [50] in the form of whole blood or centrifuged platelets. Early small trials with no control arms showed positive results [51–56]. Creaney et al. is the largest such study and compares AB with PRP finding no significant difference between the two [57]. Of AB/PRP trials using active treatments as comparators results are mixed with different methodologies [58–62]. Three RCTs comparing PRP with saline showed no effect [63–65]. Moraes’ Cochrane review of PRP identified only 19 eligible papers for inclusion and concluded there was insufficient evidence for the effect of PRP [66].

Despite much debate and a number of interventional trials and reviews it is still unclear what is the best injectable therapy to use in patients with chronic tendinopathy that has failed conservative treatment. The lack of long-term effect of CS, need for repeated injections with dextrose and polidocanol and need for centrifuge equipment combined with the strong evidence against AB/PRP point towards HVIGI as a potential superior option. However many HVIGI papers are by the same group indicating that the proposed successes may be operator dependent and to date there have been no RCTs. Frequently innovative sports medicine procedures have become commonplace without sufficient evidence and at high risk of bias [67]. The lack of properly designed RCTs relating to HVIGI and providing evidence for the separate effect of HVIGI and CS, two different mechanisms of action, needs to be addressed and is vital to further the clinical use of injectable therapies in the management of chronic tendinopathy.

## Abbreviations

AB: Autologous Blood
ADMR: Academic Department of Military Rehabilitation
ANCOVA: Analysis of covariance
ANOVA: Analysis of variance
BMI: Body mass index
CI: Chief Investigator
CPC: Clinical Policy Committee
CS: Corticosteroid
DAMOCLES: Data Monitoring Committees: Lessons, Ethics, Statistics
DDR: Director of Defence Rehabilitation
DMRC: Defence Medical Rehabilitation Centre
EL: Eccentric Loading
ESWT: Extracorporeal Shockwave Therapy
FAA: Functional Activity Assessment
GCP: Good Clinical Practice
HSR: Heavy Slow Resistance
HVIGI: High Volume Image Guided Injection
IDMC: Independent Data Monitoring Committee
IMO: Independent Medical Officer
IMP: Investigational medical product
LA: Local anaesthetic
MCID: Minimally clinically important difference
MHRA: Medicines and Healthcare Products Regulatory Agency
MO: Medical Officer
MOD: Ministry of Defence
MODREC: Ministry of Defence Research Ethics Committee
PRP: Platelet rich plasma
REC: Research Ethics Committee
RRU: Regional Rehabilitation Unit
SAEs: Significant Adverse Events
SPSS: Statistical Package for the Social Sciences
SUSARs: Suspected unexpected serious adverse reactions
US: Ultrasound
VAS: Visual Analogue Scale
VEGF: Vascular Endothelial Growth Factor
VISA-A: Victorian Institute of Sport Assessment Achilles
VISA-P: Victorian Institute of Sport Assessment Patella
